# Trends in urinary tract cancer incidence in Golestan province, Iran: a 16-year population-based study

**DOI:** 10.1186/s12885-025-14641-8

**Published:** 2025-07-28

**Authors:** Behnaz Rahatijafarabad, AmirHoushang Poorkhani, Fatemeh Esfandiari, SeyyedMehdi Sedaghat, Fatemeh Ghasemi-Kebria, Susan Hasanpour-Heidari, Saeid Amirkhanlou, Gholamreza Roshandel

**Affiliations:** 1 Golestan Research Center of Gastroenterology and Hepatology, Jorjani Clinical Sciences Research Institute, Golestan University of Medical Sciences, Gorgan, Iran; 2 Cancer Research Center, Jorjani Clinical Sciences Research Institute, Golestan University of Medical Sciences, Gorgan, Iran; 3 Department of Surgery, School of Medicine, Golestan University of Medical Sciences, Gorgan, Iran; 4 Deputy of Public Health, Golestan University of Medical Sciences, Gorgan, Iran; 5 Department of Internal Medicine, School of Medicine, Golestan University of Medical Sciences, Gorgan, Iran; 6 Sayyad Shirazi Hospital, Sayyad Shirazi Boulevard, Gorgan, Iran

**Keywords:** Kidney, Bladder, Cancer, Time Trend, Incidence

## Abstract

**Background:**

Urinary tract cancers (UTC), particularly bladder and kidney cancers, are associated with high mortality rates globally. In Iran, and specifically in Golestan province, an increasing trend in the incidence of these cancers has been reported. This study aims to analyze cancer trends and inform health policy planning to reduce the burden of these diseases in Golestan.

**Methods:**

Data was collected from the Golestan Population-Based Cancer Registry (GPCR) covering cases of UTC, particularly kidney and bladder cancer from 2004 to 2019. Crude and age-standardized rates (ASR) of kidney and bladder cancers were calculated and presented per 100,000 person-years. Estimated annual percent changes (EAPC) of the ASRs were calculated to investigate time trends in incidence rates.

**Results:**

The ASR for kidney cancer was 2.32 per 100,000, with rates of 2.75 for men and 1.91 for women. Bladder cancer had an ASR of 5.93, with 9.46 in men and 2.52 in women. Urban populations experienced higher incidence rates as 2.71 for kidney cancer and 7.01 for bladder cancer versus 1.87 and 4.80 in rural areas, respectively. The estimated annual percentage change in incidence was 3.68% for kidney and 1.56% for bladder cancer.

**Conclusion:**

Our findings suggest high rates and increasing trends in incidence of kidney and bladder cancers in Golestan. The study indicates higher kidney and bladder cancer rates in men and urban residents. Further investigations are warranted to assess the effects of risk factors like smoking, opioid drug use and agricultural factors on incidence of urinary tract cancers in this population.

## Introduction

Urinary tract cancers, which include malignant tumors of the kidney, bladder, ureter, and urethra, pose a significant global health burden by their morbidity and mortality, and their occurrence varies widely across the world. Bladder cancers consist of the majority of urinary tract cancers, while Kidney cancers are associated with the highest mortality among urinary tract malignancies [1, 2].

Bladder Cancer (BC) is the most common cancer in the urinary tract system and one of the most prevalent cancers in the world. The highest incidence rate of bladder cancer was seen in developed countries [3]. According to the International Agency for Research on Cancer (IARC) in 2022, Europe and Northern America have the highest incidence rate of BC, with age-standardized incidence rates (ASR) of 12.0 and 11.0 per 100,000 person-years [4, 5]. Worldwide, the incidence rate of BC has not changed from 1990 to 2019, which shows the diversity in the BC trends in different areas, although based on the reports, there has been a significant increase in BC ASR in East Asia. Reports reveal that Iran has followed an increasing trend of bladder cancer in recent years in both sexes. It is estimated that from 2017 to 2030, bladder cancer will continue to rise worldwide [3, 6, 7].

According GLOBOCAN-2022 estimates, kidney cancer (KC) is the 14th most common cancer worldwide with ASR of 4.4 per 100,000 person-years [4, 5]. North America and Eastern Europe have the highest incidence rate of kidney cancers, with ASR estimated as 12.6 and 10.3 per 100,000, with a higher incidence in developed countries. The ASR of kidney cancer in Western Asia was estimated as 3.9 Per 100,000 [4, 5]. In Iran, Kidney cancer was the 18th most prevalent cancer, with ASR estimated as 2.9 in males and 1.7 in females [4]. The ASR for kidney cancer has been increased worldwide from 4.72 per 100,000 in 1990 to 4.94 per 100,000 in 2017 [8].

Golestan province, situated in Northern Iran, falls within the region known as the Asian belt for esophageal cancer. Previous reports from this region suggested high incidence and increasing trends of breast and colorectal cancer and decreasing trends of esophageal cancer in this population [9, 10]. Certain lifestyle behaviors such as smoking cigarettes and hookah, using nass (a form of chewing tobacco), consuming opium, and obesity have been highlighted as related risk factors for different cancers in various reports originating from this area [11]. Many of these risk factors, mainly smoking cigarettes, are also recognized as risk factors for bladder cancer [3]. Considering the increasing trends of these risk factors, it is anticipated that urinary tract cancers will emerge as a significant health concern shortly. Understanding the demographic and geographic variations in the incidence of urinary tract cancers is essential for informing the development of effective public health policies, optimizing resource allocation, and implementing targeted cancer control initiatives.

The aim of this study is to analyze the incidence rates and temporal trends of urinary tract cancers, specifically bladder and kidney cancers, in Golestan province, Northern Iran, over a 16-year period (2004–2019), with focus on differences across regions within the province and between males and females.

## Methods

### Study design and population

The data for this study was collected from the Golestan Population-based Cancer Registry (GPCR). The GPCR covers the inhabitants of Golestan province in northeastern Iran, which spans 20,438 km2, approximately 1.3% of Iran’s total land area. Nearly half of the population of Golestan resides in rural areas, specifically in villages. The inclusion criteria for the study were individuals diagnosed with urinary tract cancers between 2004 and 2019.

### Data collection and definitions

The GPCR, a high-quality population-based cancer registry, has been a voting member of the International Association of Cancer Registries (IACR) since 2007. Its data on cancer incidence in five continents has been thoroughly reviewed and included in volumes X, XI and XII [12, 13]. Essentially, the GPCR records primary neoplasms following established standards and guidelines set by the IARC and the IACR, which includes adherence to multiple primary rules [14, 15]. Data for the registry is collected from various potential sources such as public and private hospitals, pathology centers, death registry units, and specialized medical offices within the province, with additional information obtained from cancer patients seeking treatment in neighboring cities like Tehran and Mashhad which includes 10% of patients with cancer in Golestan each year. Tumor characteristics are coded using the third edition of the International Classification of Diseases for Oncology (ICD-O-3), encompassing topography, morphology, behavior, and grade [16]. This study focuses on new cases of urinary tract cancers, including malignant tumors of the kidney, bladder, and other urinary tract organs (ICD-O-3 topography codes: C64.9-C68.9) registered by the GPCR over 16 years from 2004 to 2019.

### Statistical analysis

The data is analyzed using CanReg-5 software. The number of cases, crude rates, and Age Standardized Rates (ASRs) are calculated for the entire province population and broken down by various variables (Sex, age, place of residence, year of diagnosis, and cancer site). Age specific incidence rates were calculated in 5-years age groups. ASR is calculated using the direct standardization method and the Segi (1960) World standard population [17]. Population data for Golestan province is obtained from the statistics department of Golestan University of Medical Sciences (GOUMS), and all rates are calculated per 100,000 person-years. To assess the temporal trends in the incidence of these cancers, Estimated Annual Percent Change (EAPC) was calculated and presented for the total population based on sex and location of residence (urban versus rural areas), and its respective 95% confidence intervals (95% CI) were computed. We used the Rcan package in R program to calculate EAPCs and 95% confidence intervals for the EAPCs and incidence rates [18]. A significance level of p-value less than 0.05 was deemed statistically significant.

Ethical considerations were taken into account to protect the privacy and confidentiality of the patient’s data. The GOUMS Ethics Committee’s approval was obtained before commencing data collection and analysis to ensure adherence to ethical standards (Ethics code: IR.GOUMS.REC.1403.014).

## Results

### Overall findings

During 16 years of study period, in total 1811 urinary tract cancers were registered by GPCR which 514 (28.3%) were kidney cancer and 1269 (70%) were bladder cancer. Other and unspecified urinary organ cancers were 28 (1.5%) of the total number of cases. The number (percent) of cases who were diagnosed by pathological examination (microscopic confirmation, MV%) were 417 (81.13%) and 1107 (87.23%) for kidney and bladder cancers, respectively.

### Incidence of bladder cancer

**Fig. 1:**
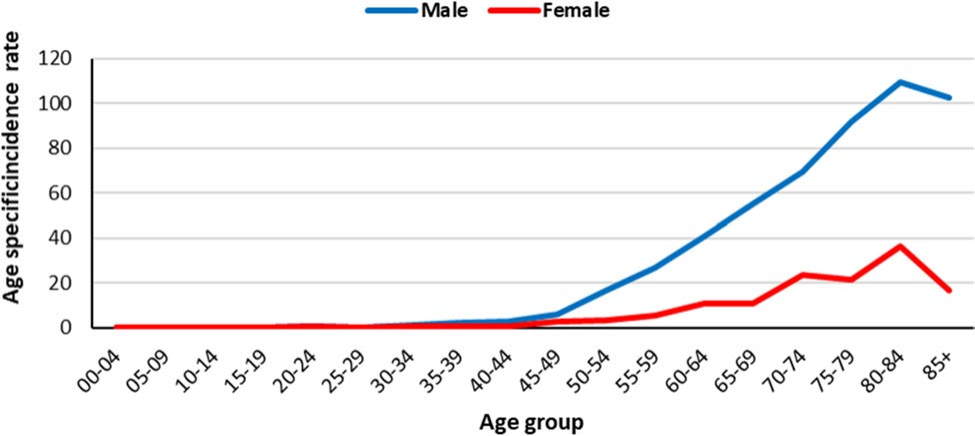
Age specific incidence rate (per 100,000 person-years) for bladder cancer in Golestan province, Iran, 2004-2019

In total 1269 cases of bladder cancer were registered by GPCR which 989 (77%) were male and 280 (22%) were female. The age-standardized incidence rate (ASR) of bladder cancer in Golestan was 5.93 (95%CI: 5.6 to 6.26) per 100,000 person-years and for males and females were 9.46 (95%CI: 8.85 to 10.07) and 2.52 (95%CI: 2.21 to 2.83), respectively. The ASR of bladder cancer is 7.01 (95%CI: 6.5 to 7.52) and 4.8 (95%CI: 4.37 to 5.23) in urban and rural areas, respectively. Table 1 shows Number of cases, crude rate, ASR and 95% confidence interval of ASR for bladder cancers by residence area and sex. Figure 1 shows Age specific incidence rate of bladder cancer between 2004 and 2019 by sex. The peak of bladder cancer rate was found in the age group of 80–84 years.

**Table 1:** Number of cases, crude rate, age-standardized incidence rate (ASR) (per 100,000 person-years) and 95% confidence interval (95% CI) of ASR for bladder cancers in Golestan province, iran, 2004–2019, by residence area and sex

Area	Sex	Number of cases	Crude rate	ASR	95% CI	
Golestan_total	Both male and female	1269	4.50	5.93	5.60	6.26
	Male	989	7.02	9.46	8.85	10.07
	Female	280	1.99	2.52	2.21	2.83
Urban area	Both male and female	776	5.35	7.01	6.50	7.52
	Male	613	8.44	11.33	10.41	12.25
	Female	163	2.25	2.79	2.34	3.24
Rural area	Both male and female	493	3.60	4.80	4.37	5.23
	Male	376	5.50	7.48	6.70	8.26
	Female	117	1.71	2.25	1.84	2.66

### Time trends of bladder cancer

The ASR of bladder cancer has an increasing trend from 4.97 (95%CI: 3.46 to 6.48) in 2004 to 7.18 (95%CI: 5.87 to 8.49) in 2019 (EAPC = 1.56%, 95% CI: −2.79 to 6.12) which was not statistically significant. The results of time trend analysis are shown in Table 2.

**Table 2:** Estimated annual percent change (EAPC) of age-standardized incidence rate (ASR) for bladder cancers in Golestan province, iran, 2004–2019, by sex and residence area

Population		EAPC (%)	95% CI	
Golestan	Total	1.56	−2.79	6.12
By sex	Male	2.62	−0.89	6.28
	Female	−1.19	−7.52	5.57
By residence area	Urban area	0.96	−3.02	5.12
	Rural area	1.79	−3.04	6.87

**Fig. 2:**
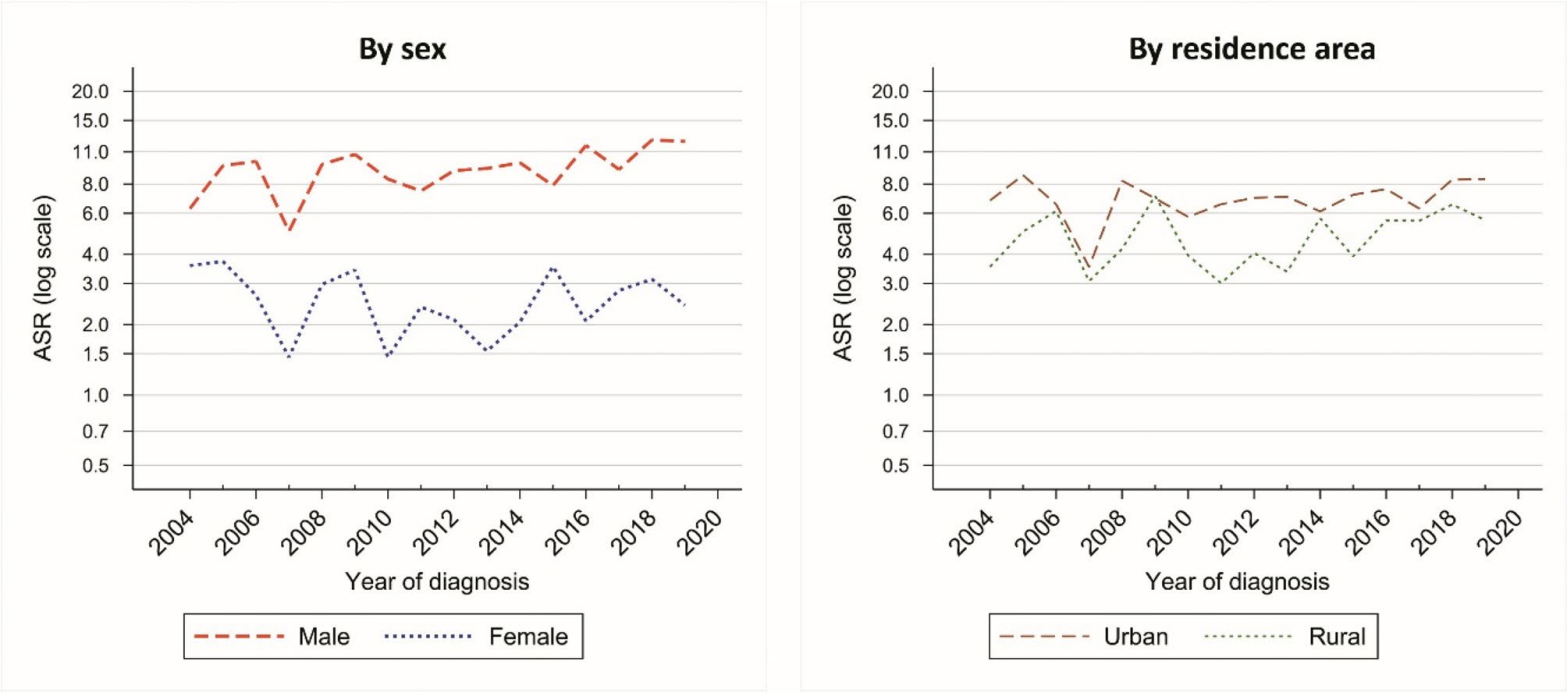
Time trends of age-standardized incidence rate (ASR) of bladder cancer in Golestan province, Iran, 2004-2019, by sex and residence area

Figure 2 shows the time trends in the ASR of bladder cancer between 2004 and 2019 by area and sex.

### Incidence of kidney cancer

**Fig. 3:**
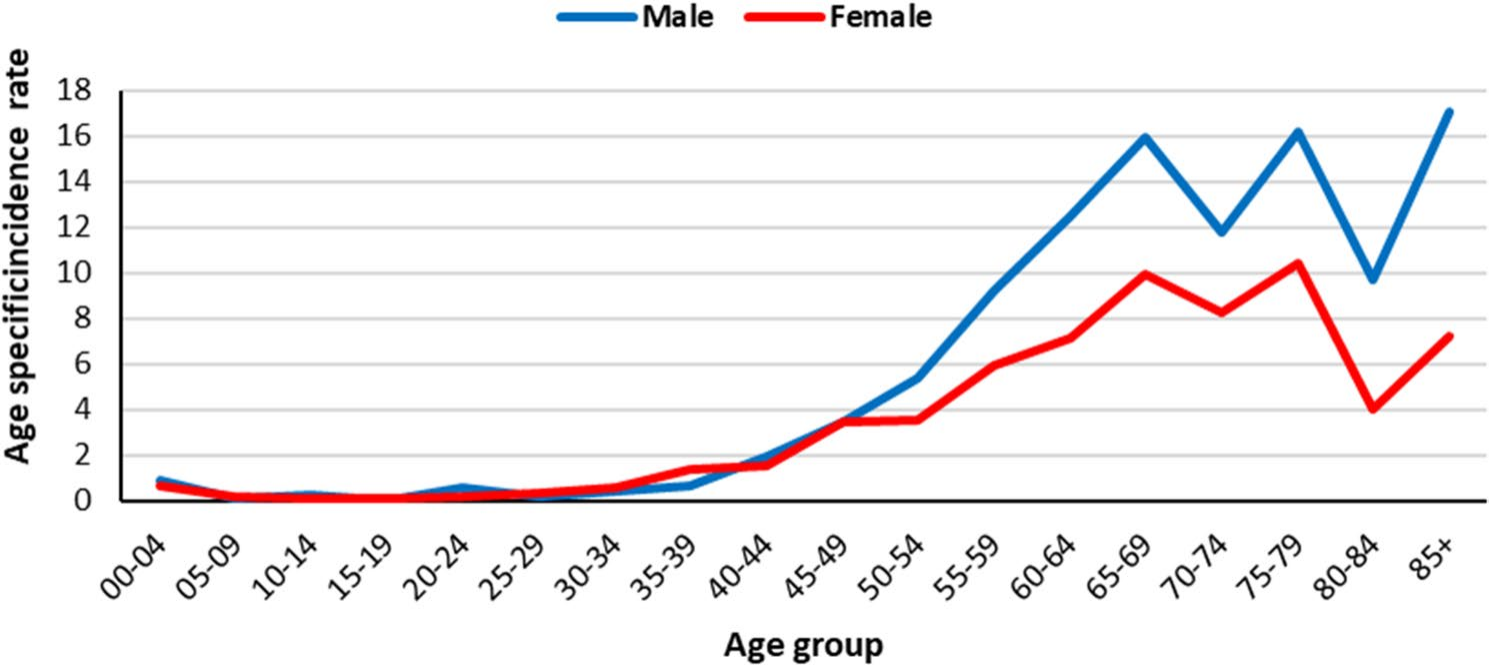
Age specific incidence rate (per 100,000 person-years) for kidney cancer in Golestan province, Iran, 2004-2019

In total 514 cases of kidney cancer were registered by GPCR which 294 (57%) were males and 220 (42%) were females. Table 3 shows ASR for kidney cancers in Golestan province between 2004 and 2019, by residence area and sex. The ASR for kidney cancer was 2.32 (95% CI: 2.1 to 2.54). Males have higher incidence of kidney cancer in Golestan province (ASR = 2.75; 95% CI: 2.42 to 3.08), compared to females (ASR 1.91; 95% CI: 1.66 to 2.16). Also, the ASR of kidney cancer in urban areas is 2.71 (95% CI: 2.4 to 3.02) compared to rural areas with ASR 1.87 (95% CI: 1.6 to 2.14). Figure 3 Shows the Age specific incidence rate of kidney cancer between 2004 and 2019 by sex.

**Table 3:** Number of cases, crude rate, age-standardized incidence rate (ASR) (per 100,000 person-years) and 95% confidence interval (95% CI) of ASR for kidney cancers in Golestan province, Iran, 2004–2019, by residence area and sex

Area	Sex	Number of cases	Crude rate	ASR	95% CI	
Golestan_total	Both male and female	514	1.82	2.32	2.10	2.54
	Male	294	2.09	2.75	2.42	3.08
	Female	220	1.56	1.91	1.66	2.16
Urban area	Both male and female	317	2.18	2.71	2.40	3.02
	Male	182	2.51	3.23	2.74	3.72
	Female	135	1.86	2.21	1.82	2.6
Rural area	Both male and female	197	1.44	1.87	1.60	2.14
	Male	112	1.64	2.20	1.77	2.63
	Female	85	1.24	1.56	1.23	1.89

### Time trends of kidney cancer

**Fig. 4:**
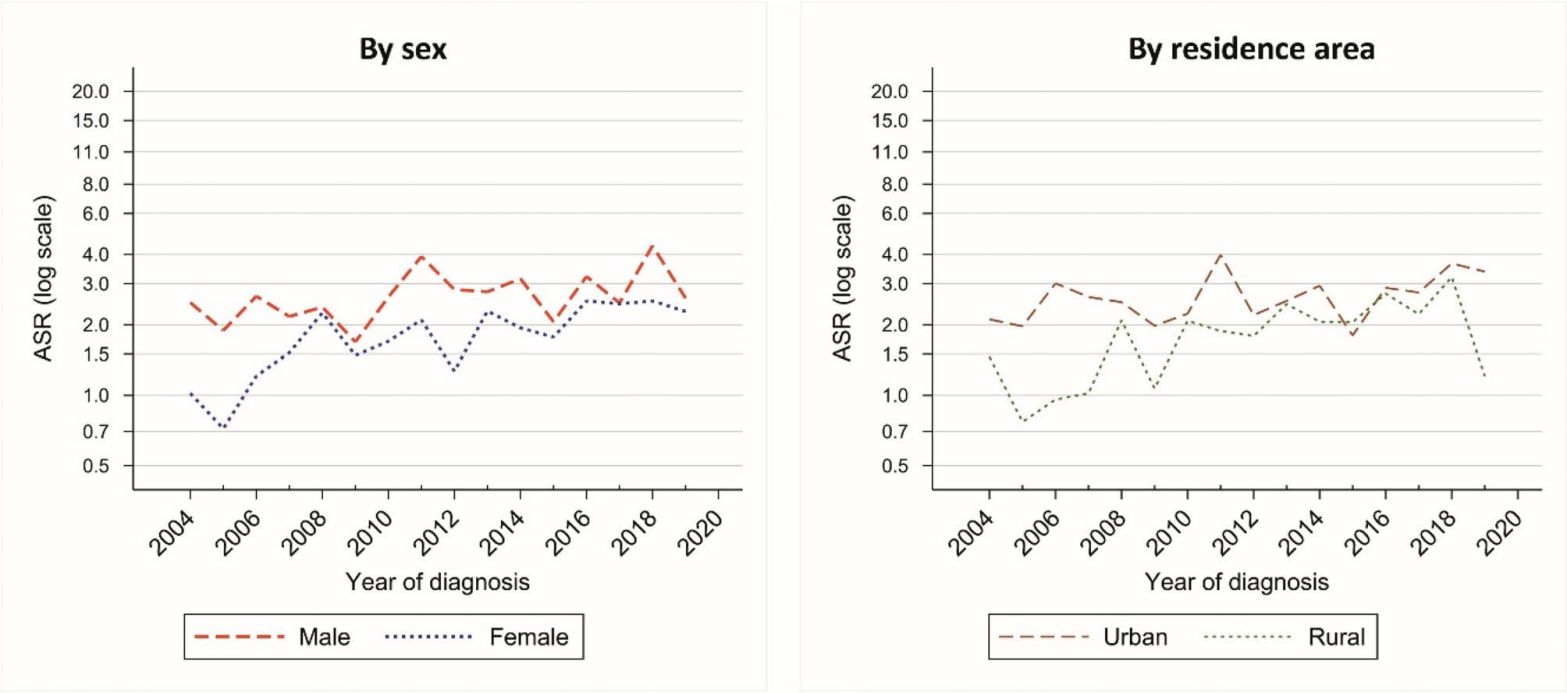
Time trends of age-standardized incidence rate (ASR) of kidney cancer in Golestan province, Iran, 2004-2019, by sex and residence area

In 2004 the ASR for kidney cancer was 1.73 (95%CI: 0.89 to 2.57) which has reached to 2.44 (95%CI: 1.7 to 3.18) by 2019 (EAPC = 3.68%; 95% CI: −3.46 to 11.36) (Table 4). Although our results did not suggest significant time trends for kidney cancer, it seems as though the increasing trends are more steep among rural communities and in females. Figure 4 shows these time trends for kidney cancer by sex and area.

**Table 4:** Estimated annual percent change (EAPC) of age-standardized incidence rate (ASR) for kidney cancers in Golestan province, Iran, 2004–2019, by sex and residence area

Population		EAPC (%)	95% CI	
Golestan	Total	3.68	−3.46	11.36
By sex	Male	2.52	−3.91	9.39
	Female	5.48	−2.65	14.30
By residence area	Urban area	2.20	−4.26	9.11
	Rural area	5.27	−2.84	14.06

## Discussion

The aim of this study was to present the incidence and time trends of urinary system cancers in Golestan province between the years 2004–2019. The age-standardized incidence rate of bladder cancer in Golestan is 5.93 per 100,000 which is 9.46 per 100,000 for males and 2.52 per 100,000 for females. According to previous studies, the incidence rate of bladder cancer in Iran in 2022 was 7.1 per 100,000. Nowroozi et al.l study has demonstrated that in Iran, the ASR for bladder cancer is 10.9 per 100,000 in males and 2.8 ASR in female [7]. The ASR for bladder cancer in the world is estimated to be 5.6 per 100,000 in 2022. This rate in Asia is 3.4 per 100,000. Golestan province, with ASR 5.93 per 100,000, has higher incidence rate of bladder cancer compared to Asia and lower rates compared to Iran.

The results of our study showed, the ASR of bladder cancer has increased from 4.97 per 100,000 in 2004 to 7.18 per 100,000 in 2019 and the EAPC of bladder cancer was 1.56%. Although our results didn’t show a significant time trend but it seems that despite the relative increase in the incidence of bladder cancer in the total population of Golestan, and especially in males, bladder cancer shows a decreasing trend in the females − 1.19%. Also the increasing trend in rural areas, is more steep than urban areas. Mousavian et al. showed, the incidence of bladder cancer in Iran has increased from 5.82 to 11.5 between 2005 and 2020. This increase is mostly due to the increase of bladder cancer in males [19],.The results of Norouzi et al. ‘s study in Iran showed that the bladder cancer’s ASR in males has increased from 8.35 to 13.57 between 2003 and 2015 in Iran. The time trends of this cancer in females have been increasing with a slow slope from 2.12 to 2.86 between 2003–2015 [7].

In 2020, the study of Chun Teoh et al. demonstrated that in European countries, the incidence rate of bladder cancer is increasing. Meanwhile, in Asia, the incidence rate of bladder cancer is decreasing [20]. Qiliang Cai and his colleagues in another study state that between 1990 and 2016, the ASR of bladder cancer has decreased from 7.11 to 6.69. This study also has showed the increase in bladder cancer incidence is mainly due to improving life expectancy and population growth in the world [6]. Considering the high incidence rate and increasing trend, this highlights the growing public health concern posed by bladder cancer in the region. In order to better understand and address this issue, it is recommended that further studies be conducted to identify the specific risk factors associated with bladder cancer in this area. Preventative measures and targeted interventions should be implemented to reduce the burden of bladder cancer in Golestan province and improve the overall health outcomes of its residents.

Among the risk factors, smoking tobacco is the greatest risk factor for bladder cancer [21]. Previous studies had showed the joint exposure to cigarette and opium has the greatest impact on these cancers [11, 22, 23]. The prevalence of monthly smoking among Iranian young adults is around 2.7–20%. Considering the high prevalence of smoking in adolescents and young adults, it is expected an increase in the incidence of bladder cancer in younger age groups in near future [24]. These findings confirm that policies to prevent and deal with the use of narcotics and especially cigarettes can play an effective role in health policies related to the control and prevention of bladder cancer.

Contact with items used in aluminum, paint, and rubber industries including aromatic amines, polycyclic aromatic hydrocarbons, and chlorinated hydrocarbons are other important risk factors for bladder cancer [3, 21]. Aromatic amines, or aryl amines are widely used in agriculture [25]. Previous studies have showed that workers in agricultural production of crops and plant farmers have a highly elevated risk for urinary bladder cancer. As agriculture is one of the main occupation in Golestan province, further investigations are warranted to assess the relationship between bladder cancer incidence and exposure to agriculture-related risk factors (e.g. pesticides) among Golestan residence.

In our study males had a higher incidence rate of bladder cancer than females, the incidence rate ratio of female to male (1:4) in bladder cancer demonstrate a significant difference in the incidence rate of bladder cancer between the two sexes. This sex disparity could be related to still high prevalence of smoking and tobacco use in males than females. Also males are more exposed to industrial and occupational chemical agents which can explain these differences [21].

The ASR of bladder cancer was 7.01 and 4.08 per 100,000 in urban and rural areas, respectively. Although our results show higher incidence rate of bladder cancer in urban areas, but previous studies in Golestan province has showed chemical agents like polycyclic aromatic hydrocarbon metabolite concentrations were almost twice as high in rural areas compared to urban areas [26]. Further studies are warranted to clarify this point.

The ASR of Kidney cancer in Golestan province is 2.32 per 100,000 which is 2.75 per 100,000 for males and 1.91 per 100,000 for females. This rate is lower compared to the ASR for kidney cancer in the world (4.4 per 100,000) and higher than Asia (2.6 per 100,000), and similar to the rates reported for Iran (2.3 per 100,000) [27, 28].

Our findings showed increasing trends in incidence rates of kidney cancer in Golestan province from 1.73 in 2004 to 2.44 in 2019, although the trend was not statistically significant. It seems that the trends in incidence rate of kidney cancer in females and rural areas are more steep than males and urban areas. Kidney cancer has faced a global increasing trend in recent years. These increasing time trends are most visible in America and developed European countries. This increase is also evident in Iran’s neighboring countries such as Turkey [27, 28]. The overall increasing trends of kidney cancer could be a result of increasing the prevalence of its risk factors related to urbanization and westernization including increasing prevalence of central obesity and metabolic syndrome. The increasing prevalence of obesity and metabolic syndrome specially in young adults could explain the shifting pattern of kidney cancer to younger age groups as we have also seen in our study [29]. Given the relatively high incidence rates and increasing trend of kidney cancer in Golestan Province, it is recommended that further studies be conducted to identify the factors associated with these changes and preventive methods in the province.

Our results have demonstrated that males (2.09 per 100,000) had a higher incidence rate of kidney cancer compared to females (1.56 per 100,000) in Golestan, which is probably related to the different prevalence of risk factors for kidney cancer among males and females. There are multiple risk factors related to kidney cancer including obesity, smoking, hypertension, Hypercaloric diet, low physical activity and exposure to Chemical Carcinogens like acrylamide, Cadmium, Arsenic and Asbestos [1, 30]. Study of Andreotti et al. has demonstrated significant elevated risks of RCC for agriculture workers in contact with herbisides and insecticides [31]. Smoking, opium use and nass chewing in males and females is highly associated with cancer rates in Golestan province. Alcala et al. has demonstrated that the fraction of cancers that were attributable to smoking cigarettes were higher among males (12%) than females (2%) and among ever opium users (21%) than never opium users (3%) in people diagnosed with cancer in Golestan province [22, 26].

As we discussed earlier hypertension is one of the risk factors for the kidney cancer. The study of Sepanlou et al. results have showed that Golestan province has a high prevalence of hypertension. This study demonstrate that males had lower blood pressure compare to females at all ages and also Turkmen, non-married and illiterate subjects, non-smokers and not opium users also showed higher systolic blood pressure [32]. Also, Previous studies have shown Golestan has a higher incidence of kidney dysfunction compared to other provinces in Iran. In 2019 Golestan has one of the highest age-standardized DALY and age-standardized death rate (ASDR) in Iran for kidney dysfunction disease which can be a risk factor for cancer [33].

Our findings suggested the age-standardized incidence rate of kidney cancer in urban areas (2.71 per 100,000) was higher than in rural dwellers (1.87 per 100,000). Low physical activity and inappropriate Western diet are other risk factors related to kidney cancer which are more common in urban area residents [32]. Zheng et al. in their study has discussed the higher prevalence of RCC in urban areas and its association with increased life expectancy in this population [34]. Golestan province has a higher level of heavy metals concentration in diet and especially in rice which residents consume, including cadmium (Cd), lead (Pb), arsenic and zinc (Zn). Consumption of rice containing high concentrations of these heavy metals could increase the risk of kidney cancer [35]. Further studies are recommended to clarify the associations between these risk factors and the risk of Kidney cancer in our population.

In our study, the highest age-specific incidence of kidney cancer was observed in females in the age range of 75–79 years and in males over 85 years of age. A previous study by Mousaviyan et al. has showed the peak age of kidney cancer in females has decreased over 15 years from 75 + years in 2005 to 74 − 65 in 2020, which has a relatively similar pattern in males [19]. It can be concluded that in the coming years, we will see a decrease in the peak age of kidney cancers in Golestan province, which requires close monitoring and pre-planned health policies.

While this study provides valuable insights into the trends of kidney and bladder cancers in Golestan province, it has certain limitations. The most important limitation is the lack of data on individual risk factors in the GPCR. Because of this, we were not able to assess the role of key contributors such as smoking, opioid use, or other lifestyle-related factors in the development of urinary tract cancers. We also could not examine the possible impact of occupational and environmental exposures, particularly in areas with higher incidence rates. Future studies are suggested to explore how lifestyle, occupational, and environmental risk factors contribute to urinary tract cancers in Golestan. Although some trends were observed in our findings, several of them did not reach statistical significance. This lack of significance introduces a level of uncertainty in interpreting the findings. It is important to consider the potential implications of these results within the context of their limitations, and further research is recommended to better understand the observed patterns.

## Conclusion

In conclusion, our results indicate relatively high and increasing incidence rates of kidney and bladder cancers in Golestan province between 2004 and 2019, with clear differences based on sex and area of residence. While the overall trends show a rise in both cancers, a slight decrease in bladder cancer incidence was observed among women. The growing trends in rural areas, compared to urban settings, are particularly concerning and emphasize the need for focused monitoring and health interventions.

Further studies are warranted to examine the contributions of known risk factors such as tobacco use, opium consumption, and agricultural exposures to the rising burden of urinary tract cancers in this population. Implementing tobacco control programs, public health education campaigns, and promoting safer farming practices could serve as practical strategies. Additionally, due to the absence of individual-level risk factor data in this study, future research should explore lifestyle, occupational, and environmental determinants, particularly in high-incidence areas, to inform more effective public health policies and cancer control efforts in the region.

## Data Availability

The datasets used and/or analyzed during the current study are available from the corresponding author on reasonable request.

## Abbreviations

UTC: Urinary Tract Cancers
GPCR: Golestan Population-Based Cancer Registry
ASR: Age-Standardized Rates
EAPC: Estimated Annual Percent Changes
IARC: International Agency for Research on Cancer
KC: Kidney Cancer
BC: Bladder Cancer
ICDO: International Classification of Diseases for Oncology
GOUMS: Golestan University of Medical Sciences
CI: Confidence Intervals
PAR: Population Attributable Risk
